# Novel Antidepressant Mechanism of Ginsenoside Rg1 in Regulating the Dysfunction of the Glutamatergic System in Astrocytes

**DOI:** 10.3390/ijms24010575

**Published:** 2022-12-29

**Authors:** Ningning Zhang, Hong Jiang, Huiqin Wang, Yating Wang, Ye Peng, Yangbo Liu, Congyuan Xia, Xu Yan, Shifeng Chu, Yi Zhang, Zhenzhen Wang, Naihong Chen

**Affiliations:** 1 State Key Laboratory of Bioactive Substances and Functions of Natural Medicines, Institute of Materia Medica & Neuroscience Center, Chinese Academy of Medical Sciences and Peking Union Medical College, Beijing 100050, China; 2 School of Pharmacy, Hunan University of Traditional Chinese Medicine & Hunan Engineering Technology Center of Standardization and Function of Chinese Herbal Decoction Pieces, Changsha 410208, China; 3 Department of Anatomy, School of Chinese Medicine, Beijing University of Chinese Medicine, Beijing 102488, China

**Keywords:** astrocytes, Cx43, ginsenoside Rg1, depression, glutamate, gap junction channel, hemichannel

## Abstract

Ginsenoside Rg1, a traditional Chinese medicine monomer, has been shown to have antidepressant effects. We previously found that Rg1 exerts antidepressant effects by improving the gap junction channels (GJCs) dysfunction; however, the downstream mechanisms through which Rg1 ameliorates GJC dysfunction remain unclear. Since hemichannels directly release glutamate, GJC dysfunction decreases the expression levels of glutamate transporters in astrocytes, and glutamatergic system dysfunction plays an essential role in the pathogenesis of depression. The glutamatergic system may be a potential downstream target of Rg1 that exerts antidepressant effects. Therefore, in this study, we aimed to determine the downstream mechanisms by which Rg1 ameliorated GJC dysfunction and exerted its antidepressant effects. Corticosterone (CORT) is used to mimic high glucocorticoid levels in patients with depression in vitro. Primary cortical astrocytes were isolated and phosphorylation of connexin43 (Cx43) as well as the functions of hemichannels, GJCs, and the glutamatergic system were evaluated after drug treatment. Rg1 pretreatment reversed the anomalous activation of Cx43 phosphorylation as well as the dysfunction of hemichannels, GJCs, and the glutamatergic system induced by CORT. These results suggest that Rg1 can ameliorate CORT-induced dysfunction of the glutamatergic system in astrocytes by potentially reducing Cx43 phosphorylation and inhibiting opening of hemichannels, thereby improving GJC dysfunction.

## 1. Introduction

Major depressive disorder (MDD), one of the most common mental disorders affecting over 350 million people worldwide, is a leading cause of disability [1]. MDD also incurs considerable economic costs and is a huge burden on society [2]. The pathogenesis of MDD is complex. Current antidepressant drugs are only effective in 60–70% of patients with depression and have many shortcomings, such as the delayed onset of action and substantial side effects [3]. Traditional Chinese medicine has unique advantages in treating depression because of its broad therapeutic targets and few side effects. Currently, several phytomedicines and their constituents are being used for the prevention and management of depression [4]. Further identification of their antidepressant mechanisms will facilitate the use of these antidepressant drugs and plants in clinical settings, and enhance our understanding of depression.

Depression was originally thought to be a neuropsychiatric disease whose root cause was neuronal dysfunction. However, evidence from the past two decades has highlighted the vital role of astrocytes in contributing to the pathophysiology and pathogenesis of depression [5]. Connexin expression and gap junctional intercellular communication are critical for astrocyte function [6]. There is direct evidence that gap junction dysfunction contributes to the pathogenesis of depression [7]. Astrocytes are highly interconnected through gap junction channels (GJCs), which facilitate the direct passage of small molecules such as ions, secondary messengers, nucleotides (ATP and ADP), and metabolites, forming an astrocytic syncytial network [8]. GJCs are formed by connexins, and connexin43 (Cx43) is the major subunit of astrocytic GJCs [8]. Apart from GJCs, connexin has been reported to form hemichannels in vitro and in vivo, promoting the direct release and uptake of neuroactive molecules, such as gliotransmitters [9]. Connexin phosphorylation is an important regulator of GJC function [6]. The functions of hemichannels are affected by many factors, including connexin phosphorylation [10]. Under physiological conditions, GJCs remain open and hemichannels remain closed [11]. Under pathological conditions, abnormally opened hemichannels release glutamate into the synaptic cleft [12], and GJC dysfunction decreases the expression of glutamate transporter GLT-1 [13], which is responsible for the uptake of glutamate from the synaptic cleft into astrocytes [5,14]. Accumulation of glutamate in the synaptic cleft damages the postsynaptic neurons and may further induce glutamatergic system dysfunction in astrocytes, leading to depression [5,15]. Therefore, the glutamatergic system may be a critical downstream target of gap junction dysfunction, which promotes the occurrence of depression.

Panax ginseng C. A. Meyer has been reported to regulate stress in the Compendium of Materia Medica. Ginsenoside Rg1 (Rg1) is the main medicinal ingredient isolated from ginseng that is effective in the prevention and treatment of depression [16]. Previous studies have shown that Rg1 exerts antidepressant effects by improving GJC dysfunction, primarily by increasing the Cx43 levels [16,17]. Apart from physically increasing the number of GJCs induced by the increase in Cx43 expression, regulation of GJC function and downstream changes are equally critical. Rg1 has been reported to improve GJC dysfunction by reducing phosphorylated-Cx43 expression at Ser368 site [18]. However, the downstream mechanisms by which Rg1 ameliorates GJC dysfunction remain unclear. Thus, in the present study, we evaluated the functional indicators of the glutamatergic system to explore their downstream mechanisms and complementarily assessed the levels of phosphorylated Cx43 at Ser279 and Tyr265 sites and the opening degree of hemichannels to determine the mechanism by which Rg1 exerts its antidepressant effects by improving GJC dysfunction.

## 2. Results

### 2.1. Purity of Cultured Rat Non-Prefrontal Cerebral Cortical Astrocytes

**Figure 1:**
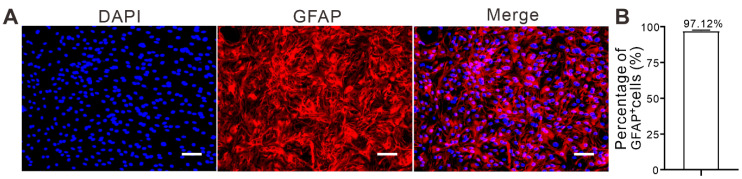
Purity of rat primary non-prefrontal cerebral cortical astrocytes. (**A**) Representative images of GFAP immunostaining in astrocytes. Red fluorescence indicates GFAP-positive astrocytes. Blue fluorescence indicates nuclei. (**B**) The ratio of GFAP-positive cells to nuclei represents the purity of astrocytes. The experiment was repeated three times, and more than 95% of cells were GFAP-positive. Scale bar: 75 µm. GFAP: Glial fibrillary acid protein.

To determine the purity of cultured astrocytes, we performed glial fibrillary acidic protein (GFAP) immunofluorescence staining (Figure 1A). Astrocytes were labeled with an anti-GFAP antibody, and cell nuclei were labeled with Hoechst 33258. The ratio of GFAP fluorescence (red) to the total number of nuclei (blue) showed that approximately 97% of the cells were GFAP-positive (Figure 1B). These cells were used for further experiments.

### 2.2. Rg1 Ameliorates CORT-Induced GJC Dysfunction of Astrocytes

**Figure 2:**
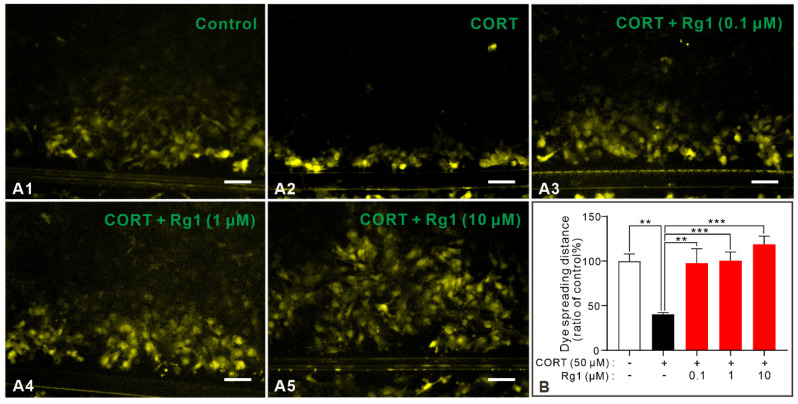
Rg1 ameliorated GJC dysfunction of astrocytes induced by CORT. (**A1**–**A5**) A scrape loading and dye transfer assay was used to assess the transfer of fluorescent Lucifer Yellow into contiguous non-prefrontal cerebral cortical astrocytes. Representative images of fluorescence captured in each group are shown. (**B**) Quantification of the fluorescence diffusion distance was performed using image analysis and expressed as percentages of the control values. CORT treatment decreased the distance of fluorescence dye diffusion, and all three concentrations (0.1, 1, and 10 μM) of Rg1 pretreatment reversed the effect. Data are shown as mean ± SEM (*n* = 8). Scar bar: 75 µm. ** *p* < 0.01; *** *p* < 0.001. CORT, corticosterone; GJC, gap junction channel.

GJCs are the fundamental structures of astrocytes. Previous studies have demonstrated that Rg1 exerts its antidepressant effects by improving GJC dysfunction. First, we investigated whether three different concentrations of Rg1 (0.1, 1, and 10 μM) could improve GJC dysfunction. CORT was used to simulate high glucocorticoid levels in patients with depression and induce GJC dysfunction. Scrape-loading and dye transfer (SLDT) is an acknowledged method for evaluating the functions of GJCs represented by the dye spreading distance. The dye spreading distance of the astrocytes in each group is shown in Figure 2. The distance of fluorescence dye diffusion was reduced in the CORT group compared to that in the control group, suggesting GJC dysfunction of astrocytes in the CORT group. All three concentrations of Rg1 significantly inhibited this reduction, implying that Rg1 can improve CORT-induced GJC dysfunction (*p* = 0.0004, F_(4,35)_ = 6.847), which is consistent with previous studies [17].

### 2.3. Rg1 Inhibits CORT-Induced Abnormal Activation of Hemichannels

**Figure 3:**
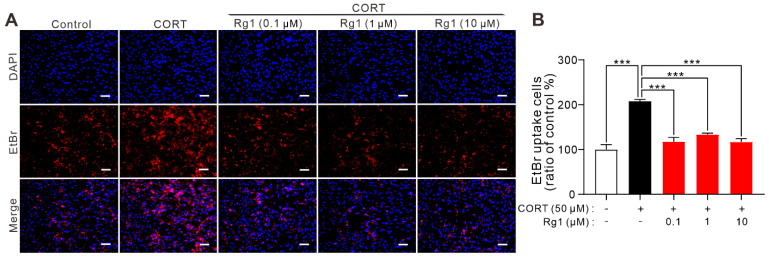
Rg1 inhibited the opening of hemichannels in astrocytes induced by CORT. (**A**) Representative images describing EtBr uptake via hemichannels in each group. Red fluorescence indicates EtBr-positive astrocytes. Blue fluorescence indicates nuclei. (**B**) The ratio of EtBr-positive cells to nuclei represents the degree of EtBr uptake in astrocytes. CORT treatment increased astrocytic EtBr uptake, and all three concentrations of Rg1 (0.1, 1, and 10 μM) pretreatment reversed the effect. All data are shown as mean ± SEM (*n* = 3). Scar bar: 75 µm. *** *p* < 0.001. CORT, corticosterone; EtBr, Ethidium bromide.

GJCs are formed by the coupled hemichannels, which can also exist independently, promoting the direct release and uptake of neuroactive molecules such as gliotransmitters [5]. Notably, Cx43 hemichannel activation induces GJC dysfunction by promoting the disintegration of GJCs [19]. Therefore, we speculated that the opening of hemichannels may be one of the mechanisms by which Rg1 ameliorates depression. Ethidium bromide (EtBr) uptake is considered as a functional index of hemichannel activity [20]; therefore, we examined the EtBr uptake levels of astrocytes in each group to evaluate the activities of hemichannels. As shown in Figure 3, CORT significantly increased the uptake of EtBr dye, indicating the increased opening of hemichannels. Furthermore, astrocytes in the (CORT + Rg1) group exhibited significantly weaker EtBr uptake than those in the CORT group (*p* < 0.0001, F_(4,10)_ = 29.78; Figure 3A,B), suggesting that the three concentrations of Rg1 reduced the opening of hemichannels induced by CORT.

### 2.4. Rg1 Reverses CORT-Induced Increase in Phosphorylated-Cx43 Levels

**Figure 4:**
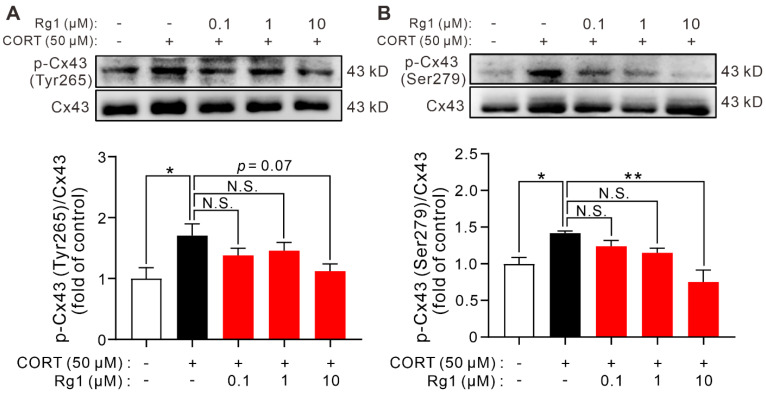
Rg1 inhibited the increase of phosphorylated Cx43 in astrocytes induced by CORT. (**A**) Regarding the major connexin in astrocytes, Cx43, CORT treatment increased the ratio of Tyr265-phosphorylated Cx43 to Cx43, and Rg1 (10 μM) pretreatment reversed this effect. (**B**) CORT treatment increased the ratio of Ser279-phosphorylated Cx43 to Cx43, and Rg1 (10 μM) pretreatment reversed this effect. All data are shown as mean ± SEM (*n* = 3). * *p* < 0.05; ** *p* < 0.01. CORT, corticosterone; N.S., no significant differences.

Cx43 is the major connexin in astrocytes, and its phosphorylation is vital for regulating GJC function. Cx43 hemichannel opening can be controlled by the phosphorylation of Cx43. To identify the mechanism underlying Rg1-improved GJC function, we extracted the total cellular protein from cultured astrocytes and performed western blotting to semi-quantitatively measure the phosphorylated-Cx43 levels (Figure 4). The ratio of p-Cx43 (Tyr265) to Cx43 in the CORT group was significantly higher than that in the control group, and this effect was reversed by Rg1 pretreatment (10 μM; *p* = 0.0591, F_(4,10)_ = 3.257; Figure 4A). Similarly, the ratio of p-Cx43 (Ser279) to Cx43 was increased by CORT treatment, and this effect was reversed by Rg1 pretreatment (10 μM; *p* = 0.0066, F_(4,10)_ = 6.774; Figure 4B).

### 2.5. Rg1 Ameliorates CORT-Induced Dysfunction of the Glutamatergic System

**Figure 5:**
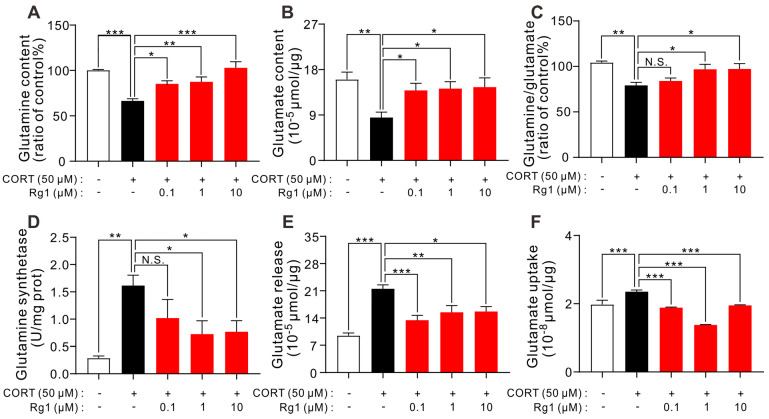
Rg1 ameliorated dysfunction in astrocytes induced by CORT. (**A**–**C**) CORT incubation significantly reduced total glutamine content (**A**), glutamate content (**B**), and the ratio of glutamine to glutamate (**C**) in astrocytes, and Rg1 pretreatment (1 and 10 μM) reversed all of these decreases. (**D**) Rg1 pretreatment (1 and 10 μM) reversed the increase of glutamine synthetase activities induced by CORT. (**E**) CORT incubation significantly increased glutamate release in astrocytes, and the increase was reversed by Rg1 (0.1, 1, and 10 μM) pretreatment. (**F**) The increased glutamate uptake induced by CORT was decreased by Rg1 pretreatment of all three concentrations (0.1, 1, and 10 μM). All data are shown as mean ± SEM (*n* = 6). * *p* < 0.05; ** *p* < 0.01; *** *p* < 0.001. CORT, corticosterone; GS, glutamine synthetase; N.S., no significant differences.

CORT incubation induces hemichannel opening and GJC dysfunction. Hemichannels directly release glutamate into the synaptic cleft under stress [21], and GJC dysfunction leads to a decrease in the expression of GLT-1 which can take up glutamate [13]. Thus, disturbance of the glutamatergic system may be a downstream mechanism through which GJC dysfunction induces depression. In fact, our previous experiments demonstrated that CORT induces the glutamatergic system dysfunction (unpublished data). Therefore, in this study, we investigated whether Rg1 could ameliorate glutamatergic system dysfunction in astrocytes. As shown in Figure 5, Rg1 reversed the downregulation of total glutamine levels (*p* < 0.0001, F_(4,25)_ = 11.46; Figure 5A), glutamate content levels (*p* = 0.0130, F_(4,25)_ = 3.934; Figure 5B), and the ratio of glutamine to glutamate (*p* = 0.0016, F_(4,25)_ = 6.010; Figure 5C) induced by CORT. Moreover, Rg1 (1 and 10 μM) ameliorated the increase in glutamine synthetase (GS) activity induced by CORT (*p* = 0.0060, F_(4,25)_ = 4.651; Figure 5D). Moreover, CORT-induced increase in glutamate release and uptake was reversed by Rg1 (0.1, 1, and 10 μM) treatment (glutamate release, *p* < 0.0001, F_(4,25)_ = 11.94 (Figure 5E); glutamate uptake, *p* < 0.0001, F_(4,25)_ = 32.72 (Figure 5F)). These data reveal that Rg1 ameliorated the glutamatergic system dysfunction induced by CORT.

## 3. Discussion

**Figure 6:**
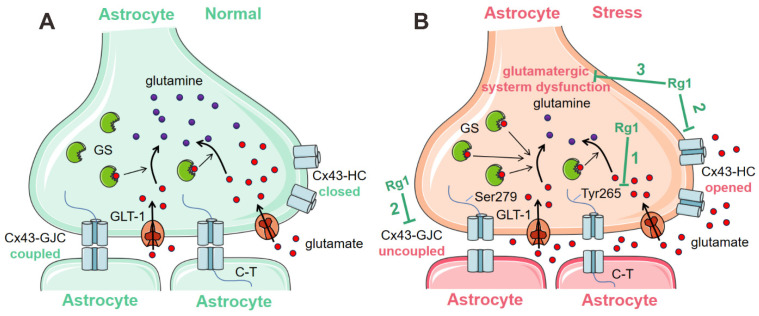
Model schematic showing the antidepressant mechanism of Rg1 based on the improvement of GJC function. (**A**) Under physiological conditions, astrocytic Cx43 is expressed in the plasma membrane and assembled into hemichannels that remain closed. Hemichannel–hemichannel interactions induce the formation of GJC between adjacent astrocytes, which allows the exchange of ions and small molecules. Over 90% of the glutamate in the synaptic cleft is taken up by GLT-1 into astrocytes to maintain a low concentration in the synaptic cleft, and then is converted to less toxic glutamine by astrocyte-specific GS. (**B**) (1) CORT induces increased Ser279-phosphorylated and Tyr265-phosphorylated Cx43, which mainly contribute to the dysfunction of GJCs. Rg1 can reverse the increase of these two phosphorylated-Cx43. (2) Increased phosphorylated-Cx43 induced the dysfunction of GJCs. CORT induces the opening of hemichannels, as well, which promotes GJC disassembly further. Rg1 can inhibit hemichannel opening and ameliorate GJC dysfunction. (3) The glutamate concentration in the synaptic cleft increases due to the opening of hemichannels. Excess glutamate in the synaptic cleft results in impaired function of the astrocyte glutamatergic system, manifested by decreased total glutamate content, decreased total glutamine content, an increase in GS activities, and an increase in glutamate release and uptake. Rg1 is able to ameliorate glutamatergic dysfunction, including all of these changes. Abbreviations: CORT, corticosterone; C-T, C-terminals; GJC, gap junction channel; GS, glutamine synthetase; HC, hemichannel.

Rg1 exerts antidepressant effects by improving the dysfunction of GJCs. However, most studies have mainly focused on the mechanism by which Rg1 increases the levels of Cx43. The present study elucidates the antidepressant mechanism of Rg1 based on GJC function. We used CORT to stimulate the stress conditions and observed the effects of Rg1 on the upstream and downstream mechanisms of GJC function regulation. As shown in Figure 6, our data revealed that Rg1 improved GJC dysfunction by decreasing p-Cx43/Cx43 levels. Additionally, Rg1 improved the function of the glutamatergic system, including glutamate release, total glutamate and glutamine content, GS activity, and glutamate uptake, by inhibiting hemichannel opening.

In this study, we selected non-prefrontal cerebral cortical astrocytes as research subjects. The cerebral cortex, the neocortex, is an advanced structure of the brain unique to mammals that is involved in the response and regulation of emotions [22]. Many neuroimaging studies have demonstrated that the cerebral cortex is thinned in patients with MDD [23], while it is thicker in patients with dysthymic disorder (DD) [24]. Antidepressant treatment can normalize cortical thickness in patients with MDD and DD [24]. The severity and clinical course of depression may depend on whether an individual is able to produce an adaptive neuroplastic response in the brain to stress [24]. Defining the molecular and cellular basis of neuroplastic changes in the cortex and identifying ways to enhance the neuroplastic response are necessary to prevent depression. Astrocytes can regulate neural plasticity by expressing receptors and regulating neurotransmitters, such as ATP, glutamate, and γ-aminobutyric acid [25]; therefore, cortical astrocytes can be used as suitable research subjects. MDD is suggested to be a “network disease” in the brain [26,27], which is associated with altered functional and structural connections in distributed brain regions [28,29]. The neuropathology of MDD involves interruptions in interactions among different brain systems [30,31]. Cortical astrocytes may be involved in the interactions among different brain regions because they can form cellular networks and have various functions, such as support, information, integration, and conduction [11]. Cx43 gene levels are downregulated in the neocortex, cerebellar cortex, mediodorsal thalamus, and caudate nucleus of patients with MDD [32]. This suggests that Cx43 may undergo extensive alterations in multiple regions of the brains of depressed individuals. Previously, we systematically studied the changes in Cx43 levels in prefrontal cortex astrocytes under stress conditions [33]. Therefore, non-prefrontal cerebral cortical astrocytes were selected for the current study. Given that the abnormal activation of the hypothalamic-pituitary-adrenal axis is an essential mechanism for the pathogenesis of depression, CORT was used to imitate the high glucocorticoid levels in patients with depression in vitro. We chose incubation of 50 μM CORT for 24 h as the stress condition, which has been shown to be the lowest concentration that can induce GJC dysfunction without affecting the cell viability [18].

First, we found that Rg1 reversed the increased phosphorylation of Cx43 at Tyr265 and Ser279 sites induced by CORT, which could be the mechanism by which Rg1 improves the dysfunction of GJCs. Cx43 is a highly regulated integral membrane protein containing two extracellular loops, a cytoplasmic loop and cytoplasmic *N*- and *C*-termini [34,35]. *C*-terminus contains sites for post-translational modifications and interactions with other cellular proteins [34,36]. Different kinases regulate the assembly, size, and turnover of GJCs by phosphorylating different sites on Cx43 [6]. Src kinase phosphorylates Cx43 at Tyr265, thereby downregulate gap junction communication and promote gap junction disassembly [37]. Mitogen-activated protein kinase (MAPK) phosphorylates Cx43 at Ser279 site, thereby reduce the “open time” of GJCs [38]. These effects play an essential role in GJC dysfunction [6].

Second, we found that Rg1 inhibited the opening of hemichannels induced by CORT, which could be another mechanism by which Rg1 improves the dysfunction of GJCs. Hemichannels are open in cerebral ischemia and heart disease. Restraint stress also induces the opening of Cx43 hemichannels in mouse hippocampal astrocytes [39]. Seven monoamine antidepressants (fluoxetine, amitriptyline, paroxetine, imipramine, reboxetine, duloxetine, and venlafaxine) exert inhibitory effects on hemichannel activities induced by lipopolysaccharides [40]. As many pathological factors that activate hemichannels also disrupt the integrity of cellular junctions, hemichannels are speculated to be potential participants in the regulation of GJCs [41,42]. In fact, Cx43 hemichannels contribute to the disassembly of cell junctions via the modulation of intracellular oxidative status. Using the lowering extracellular Ca^2+^ model, it was shown that hemichannel opening occurred earlier than the disassembly of GJCs. Moreover, blocking hemichannels with chemical inhibitors or downregulating Cx43 expression with short interfering RNA attenuates the disruption of GJCs [19]. Interestingly, a Cx43 mutant was used to emulate Cx43 phosphorylation, and it showed hemichannel opening at normal external calcium concentrations [43]. Therefore, we speculate that Cx43 phosphorylation regulates the functions of hemichannels and GJCs and that hemichannel activation under abnormal conditions promotes GJC dysfunction. Rg1 inhibited hemichannel openings and improved GJC dysfunction partly by decreasing Cx43 phosphorylation. However, whether phosphorylation at different sites occurs independently or in tandem, transiently or continuously, and alternately or progressively remains unclear [43]. Further experiments are needed to complement the regulation of phosphorylation of Cx43 at different sites by Rg1.

Finally, our data showed that Rg1 ameliorated glutamatergic system dysfunction induced by CORT, which may be the downstream mechanism by which Rg1 exerts its antidepressant effects by improving GJC dysfunction. The release of gliotransmitter through hemichannels is a crucial function in brain physiology. Uncontrolled opening of these channels exacerbates gliotransmitter release, which is toxic to neighboring cells at high concentrations [12,44]. Abnormal hemichannel opening and release of glutamate [12] may induce glutamatergic system dysfunction in astrocytes. GJC dysfunction also decreases the expression levels of the glutamate transporter, GLT-1, in astrocytes [13]. Therefore, disturbance of the glutamatergic system may be a downstream mechanism in depression caused by the dysfunction of GJCs and hemichannels. Glutamate is the major excitatory neurotransmitter in the brain that is involved in most excitatory transmissions; however, excessive glutamate release can cause excitotoxicity and brain damage [45]. Astrocytes actively participate in glutamatergic activities, including glutamate uptake, metabolism, and recycling [46]. Astrocyte-specific glutamate transporter, GLT-1, takes up glutamate in the synaptic cleft to ensure low concentrations [5,14]. In astrocytes, glutamate is transformed to glutamine by glial-specific GS, and glutamine then shuttles back to neurons for reconversion to glutamate [11]. Therefore, glutamate, glutamine, and GS are important glutamatergic markers of astrocytes, and their abnormal changes reflect astrocyte dysfunction [11]. Here, we found that Rg1 reversed the CORT-induced decrease in glutamate and glutamine levels induced by CORT. Levels of glutamate and glutamine are significantly decreased in several brain regions, including the prefrontal cortex [47], anterior cingulate cortex [48,49], and the combined region of the amygdala and anterior hippocampus [50,51] in living subjects with MDD. Our data showed that Rg1 reversed the CORT-induced increase in GS enzyme activity. GS protein expression levels in the orbitofrontal cortex of subjects with MDD are decreased [52]. GS is highly expressed in, but not restricted to, astrocytes [53]. Although oligodendrocytes (OLs) are not involved in the glutamate-glutamine cycle, roughly half of cortical perineuronal OLs are GS-immunopositive, and the role of GS in OLs remains to be elucidated [53]. Besides, a “sister” enzyme (GS-like protein) that is very closely related to GS immunologically and enzymatically has been found in human brains [54], which may affect the glutamate–glutamine cycle. These factors may account for inconsistent changes in GS activity in vitro and in vivo under stress. We also found that Rg1 reversed the CORT-induced increase in glutamate release. Rg1 may reduce glutamate release by inhibiting hemichannel opening [11], which is the main mechanism by which Rg1 improves the function of the glutamatergic system in astrocytes. Here, we found that CORT increased glutamate uptake, and that Rg1 treatment reversed this effect. Interestingly, female rats subjected to the learned helplessness paradigm showed decreased glutamate uptake in the cortex [55]. In astrocytes, glutamate uptake in the synaptic cleft is mainly mediated by GLT-1. The blockade of astrocytic glutamate uptake in the prefrontal cortex is sufficient to produce anhedonia, a core symptom of depression [56]. We suspect that the effects of GJC and hemichannel dysfunction on the glutamatergic system may be time-dependent. In the short term, glutamate release from activated hemichannels induced by CORT can be beneficial for adaptation to environmental changes [11]. To maintain low glutamate concentrations in the synaptic cleft, glutamate uptake by astrocytes is increased. However, continuous activation of hemichannels and dysfunction of GJCs result in the accumulation of glutamate in the synaptic cleft. Prolonged stress ultimately impairs the astrocyte glutamatergic system function, in which glutamate uptake is reduced [11]. Rg1 can normalize various indicators of the glutamatergic system in astrocytes after CORT treatment, which may be an antidepressant mechanism of Rg1.

Our previous study revealed that Rg1 exerts antidepressant effects by improving GJC dysfunction and increasing Cx43 protein levels. In addition, there are other antidepressant mechanisms of Rg1 [57], including increased brain-derived neurotrophic factor (BDNF) levels [58], promotion of hippocampal neurogenesis [58], amelioration of neuroinflammation [59], oxidative stress [59], and increased synaptic plasticity [60]. Rg1 appears to improve the brain function in depression models; therefore, we speculated that these mechanisms may be associated with each other. In cortical astrocytes, downregulation of Cx43 levels increases BDNF expression levels via adrenergic receptors [61,62]. The ameliorative effect of Rg1 on neuroinflammation appears to occur partly via the inhibition of Cx43 ubiquitination [63]. Hemichannels can be activated by proinflammatory cytokines (tumor necrosis factor-α and interleukin-1β) and reactive oxygen species [64] and activated hemichannels can further release glutamate and ATP [12]. One of the signals of inflammatory activation is attributed to ATP [65,66] and glutamate via N-methyl-D-aspartate receptors [67]. Inflammation can further promote the opening of hemichannels, creating a vicious cycle that continuously promotes the inflammatory response [68]. In addition, GJCs may provide a pathway for ATP [69]. Glutamate is closely related to the regulation of neurogenesis [70] and synaptic plasticity [71]. All available data suggest that the antidepressant mechanism of Rg1 may be related to these aspects, but there is no in-depth study elucidating the connection between them. Exploring the interconnections of these mechanisms will help to better understand the pathogenesis of depression and the antidepressant mechanisms of Rg1.

We noticed a difference in the effective dose of Rg1 between different experiments. In experiments in which the functions of the glutamatergic system and GJC were measured, almost all three doses of Rg1 exerted ameliorative effects, whereas in the p-Cx43 assay, only high doses of Rg1 ameliorated the effects of CORT. This may be because of two reasons. First, the function of hemichannels is very susceptible to extracellular and intracellular microenvironments [10], including Ca^2+^ concentration outside and inside the cell, protein kinase and protein phosphatase levels, and mechanical stress [10]. Therefore, the function of the hemichannel may be sensitive to the action of Rg1, whose lowest dose (0.1 μM) significantly improved the hemichannel function. Improvement in GJC and glutamatergic system functions by Rg1 may be multifaceted. Rg1 broadly affects the functions of neurons, astrocytes, and microglia in the cerebral cortex [72] and limbic system [73,74,75]. The mechanisms involve the regulation of neuroinflammation [74], neurotransmitters [75], oxidative stress [19,59], and synaptic plasticity [60,73]. Oxidative stress [19] and neuroinflammation [76] are important factors contributing to GJC dysfunction. Second, experimental errors may have some effects. Compared to functional experiments, the whole process of western blotting is longer and more complex, which makes it more susceptible to interference and leads to a decrease in experimental sensitivity. Overall, our data tentatively suggests that Rg1 can improve the levels of p-Cx43 and the functions of hemichannels, GJCs, and the glutamatergic system. However, the effects of Rg1 dose are related to more complex antidepressant mechanisms, which need to be investigated further in future studies.

### Limitations and Future Directions

This study has some limitations. First, although the CORT model used in this study can simulate high glucocorticoid levels in depressed patients, the concentration we used (50 μM) was much higher than that observed in the serum of depressed patients (822.1 ± 43.8 nM) [77]. Therefore, it should be further validated in commonly used animal models of depression. Second, we examined the effects of Rg1 on Cx43 phosphorylation only at Ser279 and Tyr265 sites in this study; however, there are multiple phosphorylation sites on the Cx43 *C*-terminal tail, and phosphorylation at these sites has complex regulatory effects on the functions of GJCs and hemichannels [78]. Some studies have suggested that the phosphorylation of the MAPK site, Cx43S255/262/279/282A (MK4), promotes the interactions between the *C*-terminal tail and intracellular loops, bringing hemichannels into a state available for opening [79] and resulting in the closure of GJCs [80]. Abnormal phosphorylation of MK4 sites may be an important factor in reducing the dysfunction of Cx43 GJCs and hemichannels. Therefore, we should further explore the effect of Rg1 on MK4-phosphorylation to provide new insights into the mechanisms of depression.

## 4. Materials and Methods

### 4.1. Isolation and Culture of Primary Astrocytes

Primary non-prefrontal cerebral cortical astrocytes were obtained from one-day-old rats as described previously [33]. All experimental protocols were approved by the Animal Care Committee of the Peking Union Medical College and Chinese Academy of Medical Sciences (approval No. 00003981, approved on 10 August 2021). Briefly, the rats were sterilized in 75% ethanol and their heads were cut in a cold Dulbecco’s Modified Eagle’s Medium/Nutrient Mixture F-12 (DMEM/F12) (Gibco, Grand Island, NY, USA). The cerebral cortex was isolated in cold DMEM/F12 medium, the prefrontal cortex was stripped, and the remaining tissue was digested with 0.25% trypsin (0.04% EDTA) for 10 min at 37 °C. The obtained cells were centrifuged at 1000× *g* for 5 min at 4 °C. The cell pellet was washed twice with cold DMEM/F12 medium and resuspended in DMEM/F12 medium containing 15% fetal bovine serum (Gibco, Grand Island, NY, USA) and 1% penicillin and streptomycin. Finally, cells were cultured in an incubator at 37 °C and 5% CO_2_. The medium was changed every 2–3 days. After 10 days, astrocytes were purified twice by shaking at 180 rpm for 6 h, and the medium was changed after every purification step. Finally, cells were stained for immunofluorescence with the astrocyte-specific marker, rabbit anti-GFAP antibody (1:1000; Abcam, Cambridge, UK; #12389S). Astrocyte purity is defined as the ratio of GFAP-positive cells to the nucleus.

### 4.2. Cell Treatments

CORT (Sigma-Aldrich, St. Louis, MO, USA) was used to simulate high glucocorticoid conditions in vitro. Cells were incubated with CORT (50 μM) for 24 h, which has been reported to induce GJC dysfunction without affecting the cell viability [18]. Cells were randomly divided into the control, the CORT, and CORT + Rg1 groups, with three concentrations of Rg1 (0.1, 1, and 10 μM). Rg1 was pre-incubated for 1 h, followed by co-incubation with CORT for 24 h.

### 4.3. SLDT Assay

SLDT assay, a well-known method to evaluate the functions of GJCs, was used to evaluate the GJC function of astrocytes in each group, as described previously [10]. Briefly, after drug treatment, astrocytes were washed three times with phosphate-buffered saline (PBS) and scraped using a razor blade. Cells were then incubated with Lucifer yellow (1 mg/mL) for 10 min at 25 °C, washed thrice with PBS, and fixed with 4% paraformaldehyde (PFA). Dye diffusion was observed using a fluorescent microscope, and the dye diffusion distance was analyzed using Image-Pro Plus 6.0 (Media Cybernetics, Bethesda, MD, USA) to identify the functions of GJCs.

### 4.4. EtBr Uptake Assay

An uptake assay using the hemichannel-permeable reporter dye EtBr (Med Chem Express, Monmouth Junction, NJ, USA) was performed to evaluate the functions of hemichannels as previously described. EtBr is impermeable through the membrane, but can pass through hemichannels and produce fluorescence after binding to DNA. After drug treatment, astrocytes were exposed to EtBr (5 μM) for 10 min at 37 °C. Cells were washed thrice with PBS and fixed with 4% PFA for 10 min. Finally, nuclear staining was performed using Hoechst 33342 (Dojindo Laboratories, Kumamoto, Japan) for 10 min. Dye absorption was imaged via epifluorescence (518 nm excitation and 605 nm emission wavelengths) using a fluorescence microscope (Nikon, Tokyo, Japan). Data were calculated as the percentage of EtBr-positive cells in each field.

### 4.5. Glutamate Release and Uptake Assay

Glutamate release and uptake assay was modified from the one described by Shaimaa et al. [81]. Briefly, astrocytes were seeded into 96-well plates at a density of 40,000 cells/well. After drug treatment, cells were washed twice with PBS. For glutamate release, cells were incubated with artificial cerebrospinal fluid (aCSF) without glutamate for 5 h at 37 °C. Finally, the supernatant was collected and assayed for glutamate concentration using a glutamate assay kit (Abcam, Cambridge, UK) according to the manufacturer’s instructions. For glutamate uptake, astrocytes were incubated in aCSF with 200 µM glutamate for 5 h. Glutamate uptake was measured by subtracting the amount of glutamate assayed in the medium from the amount added to the cells. The supernatant was collected and assayed for glutamate concentration using a glutamate assay kit. The cell total protein content of each group was measured using a BCA protein assay kit (Applygen, Beijing, China) to normalize the glutamate levels.

### 4.6. Measurement of Intracellular Glutamine and Glutamate Levels, and GS Activity

Astrocytes were seeded into 5-mL culture flasks, and drug treatment was performed after the cells became confluent to the bottom. Cells were centrifuged at 2000× *g* for 5 min at 4 °C, and cell pellets were lysed using the lysis buffer in the corresponding kit at 4 °C. Cell homogenates were centrifuged at 12,000× *g* for 30 min at 4 °C. A glutamine assay kit (Grace Biotechnology, Suzhou, China), glutamate assay kit (Abcam, Cambridge, UK), and GS kit (Grace Biotechnology, Suzhou, China) were used to assay glutamine concentration, glutamate concentration, and GS activity in the supernatant, according to the manufacturer’s instructions. All measurement results were normalized to the corresponding cell total protein content, measured using the BCA assay.

### 4.7. Western Blotting Analysis

Western blotting was performed as previously described, with minor modifications [16]. Astrocytes were seeded into 5-mL culture flasks until they became confluent to the bottom and drug treatment was performed. Then, cells were centrifuged at 2000× *g* for 5 min at 4 °C, and cell pellets were lysed using the lysis buffer for 30 min at 4 °C and shocked every 10 min. Homogenates were centrifuged at 12,000× *g* for 30 min at 4 °C. Protein concentration was determined using a BCA protein assay kit. Equal amounts of protein were separated on 10% sodium dodecyl sulfate-polyacrylamide gels (Biomol, Hamburg, Germany) and transferred onto polyvinylidene fluoride membranes. The membranes were blocked with 5% bovine serum albumin in Tris-buffered saline for 2 h at room temperature and incubated overnight at 4 °C with the following primary antibodies: anti-Cx43 (1:1000; Cell Signaling Technology, Danfoss, MA, USA; #3512S), anti-phospho-Cx43 (Ser279) (1:2000; absin, Shanghai, China; #abs140057), anti-phospho-Cx43 (Tyr265) (1:2000, Affinity, Guangzhou, China, #AF2306), and anti-glyceraldehyde 3-phosphate dehydrogenase (1:2000; Cell Signaling Technology; #5174S) antibodies. The membranes were then washed with Tris-buffered saline containing 0.1% Tween-20, followed by incubation with secondary anti-rabbit antibodies (1:5000; KPL, Milford, MA, USA; #5220-0336) at room temperature for 2 h. Densitometry was performed using Image Quant LAS 4000 mini (Fujifilm, Tokyo, Japan), and analyzed using Quantity One 4.6.2 (Bio-Rad, Hercules, CA, USA).

### 4.8. Statistical Analysis

Data were analyzed using one-way analyses of variance, followed by Dunnett’s multiple comparison test. Statistical significance was set at *p* < 0.05. All results are expressed as the mean ± standard error of the mean. GraphPad Prism (version 8.0; GraphPad, San Diego, CA, USA) was used for statistical analysis.

## 5. Conclusions

In conclusion, our findings revealed that Rg1 can ameliorate CORT-induced dysfunction of the glutamatergic system in astrocytes. The underlying mechanisms involve reducing the phosphorylation of Cx43 and inhibiting the opening of hemichannels, thereby improving the dysfunction of GJCs. The astrocyte glutamatergic system is a downstream target of Rg1 because of its antidepressant effect, which is based on the improvement of GJC function. Therefore, this study provides novel insights into the pathogenesis of depression as well as the antidepressant mechanisms of Rg1.

## Data Availability

The data presented in this study are available on request from the corresponding author.

## Abbreviations

aCSF: artificial cerebrospinal fluid; CORT, corticosterone; Cx43, Connexin43; EtBr, ethidium bromide; GFAP, glial fibrillary acidic protein; GJCs, gap junction channels; GS, glutamine synthetase; HPA axis, hypothalamic-pituitary-adrenal axis; LY, Lucifer yellow; MAPK, mitogen-activated protein kinase; MDD, major depression disorder; OL, oligodendrocytes; PBS, phosphate-buffered saline; PFA, paraformaldehyde; Rg1, Ginsenoside Rg1; SLDT, scrape-loading and dye transfer.
